# PTP4A1 promotes oral squamous cell carcinoma (OSCC) metastasis through altered mitochondrial metabolic reprogramming

**DOI:** 10.1038/s41420-023-01657-x

**Published:** 2023-09-29

**Authors:** Bing Liu, Wen Si, Bo Wei, Xuan Zhang, Peng Chen

**Affiliations:** 1 Department of Stomatology, Air Force Medical Center of Chinese PLA, Beijing, 100142 China; 2 Department of Medical Oncology, Beijing Shijitan Hospital affiliated to Capital Medical University, Beijing, 100038 China; 3 Department of Stomatology, The First Medical Center of Chinese PLA General Hospital, Beijing, 100853 China; 4 Hospital Management Research Institute, Innovative Medicine Department Chinese PLA General Hospital, Beijing, 100853 China

**Keywords:** Cancer, Molecular biology

## Abstract

PTP4A1 (Protein tyrosine phosphatase 4A1) is a protein tyrosine phosphatase that regulates a range of pro-oncogenic signaling pathways. Here, we report a novel role for PTP4A1 in oral squamous cell carcinoma (OSCC) growth and development. We show that PTP4A1 is frequently overexpressed in OSCC cells and tissues compared to adjacent non-tumor tissue. In OSCC, the overexpression of PTP4A1 increased cell growth and invasion in vitro, and enhanced tumor progression in vivo. At the molecular level, PTP4A1 was found to regulate mitochondrial metabolic reprogramming to enhance the invasive capacity of OSCC cells. Mechanistically, these effects were mediated through binding to pyruvate kinase isoenzyme M2 (PKM2) to promote its expression and aconitase 2 (ACO2) to enhance its degradation. Together, these data reveal PTP4A1 as a viable target for OSCC therapeutics.

## Introduction

Oral squamous cell carcinoma (OSCC) is a globally prevalent cancer [1, 2] with high rates of recurrence. Despite advances in diagnostics and therapy, the survival rates of OSCC cases remains low. Surgery remains the major treatment modality [3, 4]. Understanding the molecular events that lead to OSCC progression and development can help guide the development of new and much-needed treatments.

Protein tyrosine phosphatase 4A1 (PTP4A1) is a membrane-associated protein tyrosine phosphatase that is subject to prenylation and upregulated in an array of cancers [5–7]. PTP4A1 activates PI3K/AKT signaling and is pro-oncogenic in intrahepatic cholangiocarcinoma [5] and is a critical enhancer of oncogenic TGFβ mediated signaling [8]. PTP4A1 expression is shown to be enhanced in cervical cancer cells by preventing its degradation through sponging microRNA-299-3p by the long-non-coding RNA USP30-AS1 [9]. High PTP4A1 expression is also a prognostic marker of poor survival in non-small lung cancer [10].

Cancer cells use aerobic glycolysis for adenosine triphosphate (ATP) production and display enhanced glucose uptake and lactic acid secretion to meet their energy requirements. Cancer progression can be driven by pathways that promote aerobic glycolysis within tumor cells, including mitochondrial-mediated redox signaling. Numerous studies now highlight how cancer cells enhance mitochondrial biogenesis [11] to promote malignant tumor progression [12]. Here, we show that PTP4A1 is highly-expressed in OSCC in vitro and in vivo and promotes mitochondrial metabolic reprogramming to enhance invasive capacity through enhanced PKM2 transcription and ACO2 degradation. We therefore reveal new information on the cellular role of PTP4A1 in OSCC, highlighting its potential as a therapeutic target.

## Results

### PTP4A1 is highly expressed in OSCC

**Fig. 1:**
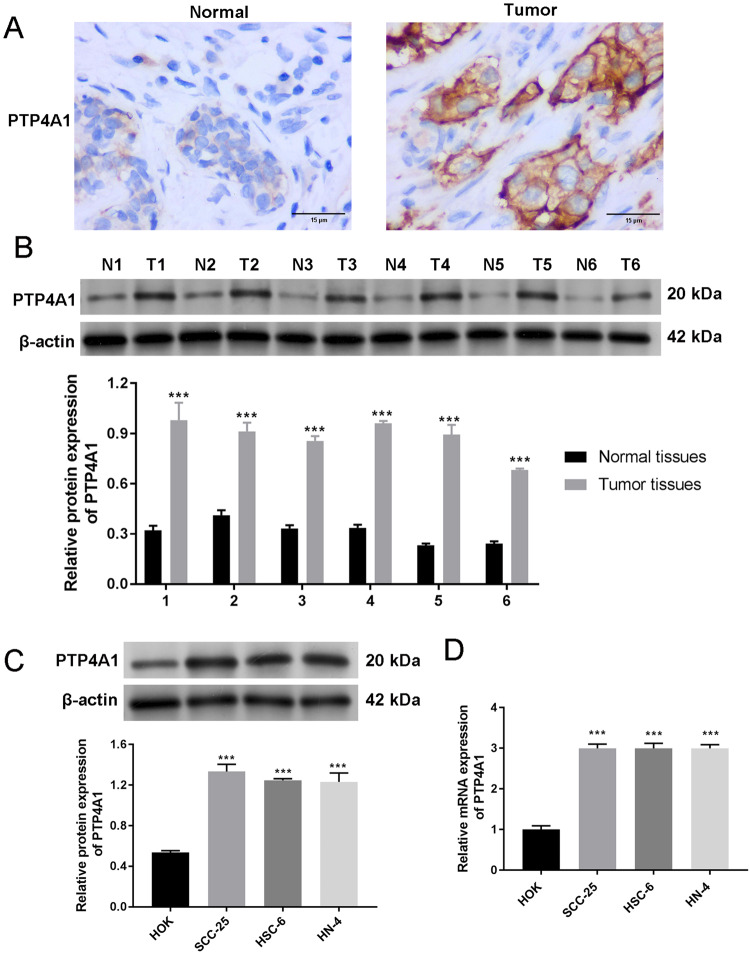
PTP4A1 is overexpressed in OSCC tissues and cells. **A** Representative Immunohistochemistry (IHC) staining for PTP4A1 in OSCC and normal tissue sections (brown, scale bar = 15 μm, magnification ×400). **B** PTP4A1 expression in 6 pairs of OSCC and matched normal tissues analyzed by western blotting. **C** PTP4A1 expression in OSCC- (SCC-25, HSC-6, and HN-4) and human oral keratinocyte (HOK) cells assessed via western blotting. **D** RT-qPCR detection of PTP4A1 expression in SCC-25, HSC-6, and HN-4 cells and human oral keratinocytes (HOK). *P < 0.05, **P < 0.01. ***P < 0.001.

IHC was performed to detect the protein expression of PTP4A1 in normal *vs*. OSCC tissues. PTP4A1 expression was upregulated in OSCC samples in comparison to healthy tissue (Fig. 1A). These data were confirmed through western blot analysis on 6 paired OSCC and normal tissue samples that showed consistently higher PTP4A1 expression in cancer tissue (*P* < 0.001, Fig. 1B). As shown in Fig. 1C, D, PTP4A1 expression was higher in SCC-25, HSC-6, and HN-4 cells compared to non-HOK cells (*P* < 0.001). These data confirmed that PTP4A1 is upregulated in OSCC.

### Knockdown of PTP4A1 suppresses OSCC metastasis

**Fig. 2:**
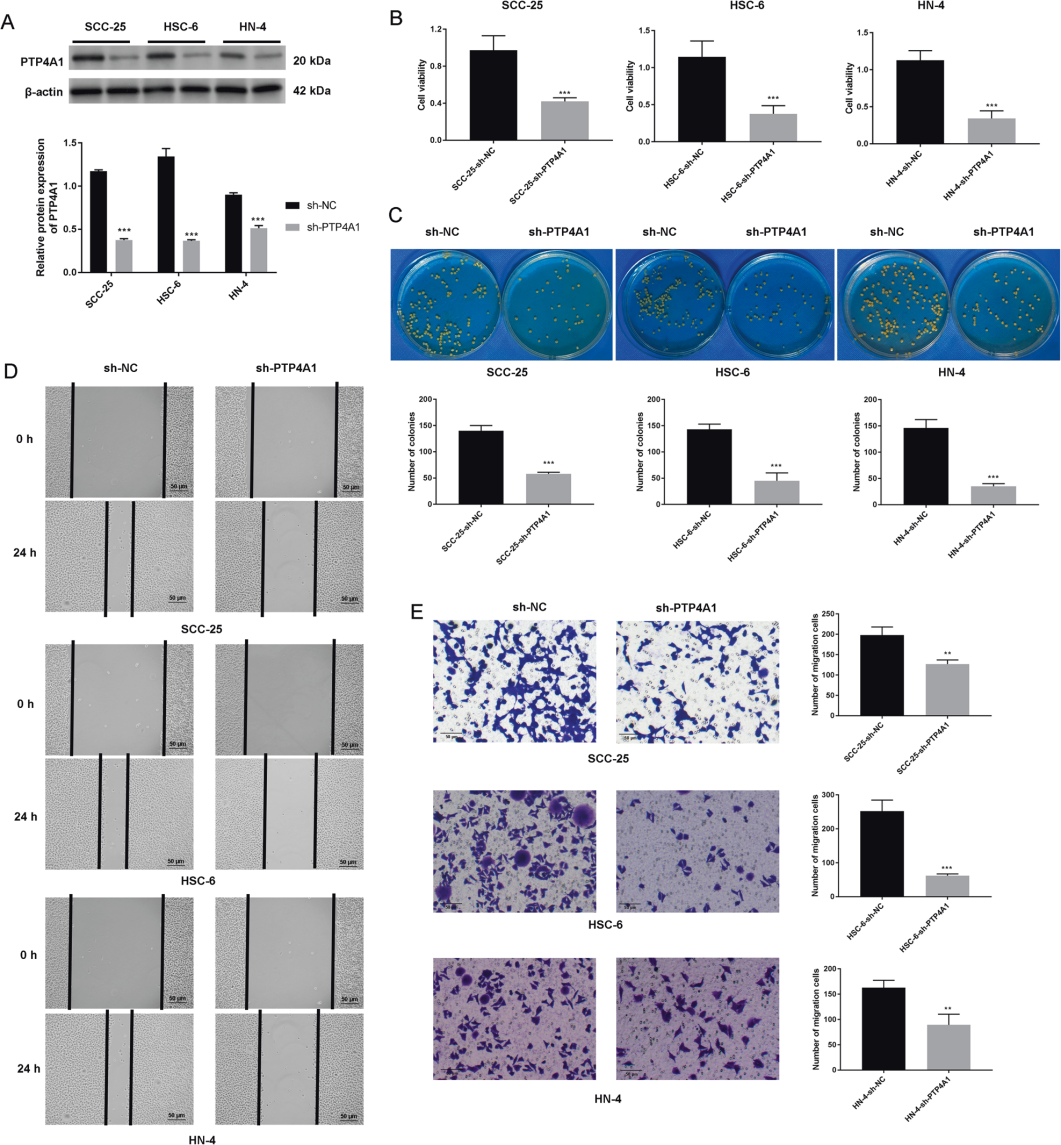
Knockdown of PTP4A1 suppresses the growth and metastasis of OSCC cells. **A** PTP4A1 expression in PTP4A1-knockdown OSCC cells (SCC-25, HSC-6, and HN-4) detected via western blotting. **B** MTT assays and (**C**) soft agar colony formation assays in PTP4A1-silenced OSCC cells. **D** sh-PTP4A1 inhibits OSCC cell migration assessed by wound healing assays. **E** sh-PTP4A1 inhibits OSCC cell invasion assessed by Transwell invasion assays. **P < 0.01, ***P < 0.001 vs. sh-NC.

We next investigated the effects of PTP4A1 silencing using an sh-PTP4A1 lentivirus. Knockdowns of PTP4A1 in sh-PTP4A1 SCC-25, HSC-6, and HN-4 cells compared with sh-NC cells were confirmed by western blotting (Fig. 2A, *P* < 0.001). MTT assays demonstrated that compared to sh-NC cells, the viability of sh-PTP4A1 cells were markedly reduced (*P* < 0.001, Fig. 2B). Similarly, colony formation assays showed that sh-PTP4A1 cells had lower levels of cell proliferation and colony forming capacity than sh-NC cells (*P* < 0.001, Fig. 2C). sh-PTP4A1 also inhibited the migration of OSCC cells in wound healing (Fig. 2D) and Transwell assays (*P* < 0.01, *P* < 0.001, Fig. 2E).

### Knockdown of PTP4A1 induces mitochondrial metabolic reprogramming in OSCC cells

**Fig. 3:**
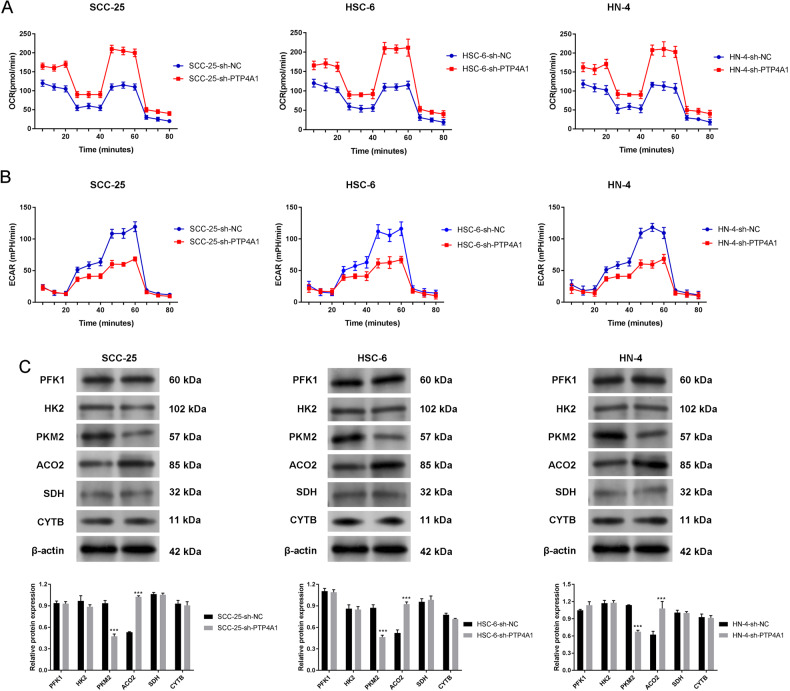
Knockdown of PTP4A1 induces mitochondrial metabolic reprogramming in OSCC cells. **A** Seahorse assay demonstrating increased oxidative consumption rates (OCR) in sh-PTP4A1 cells compared to sh-NC in response to oligomycin. **B** Seahorse assays demonstrating the decreased extracellular acidification rate (ECAR) in sh-PTP4A1 cells compared to sh-NC in response to oligomycin. **C** Expression of key glycolytic enzymes (PFK1, HK2, and PKM2) and key oxidative phosphorylation enzymes (ACO2, SDH and CYTB) in transfected OSCC cells (SCC-25, HSC-6, and HN-4) detected by western blotting. ***P < 0.001 vs. sh-NC.

**Fig. 4:**
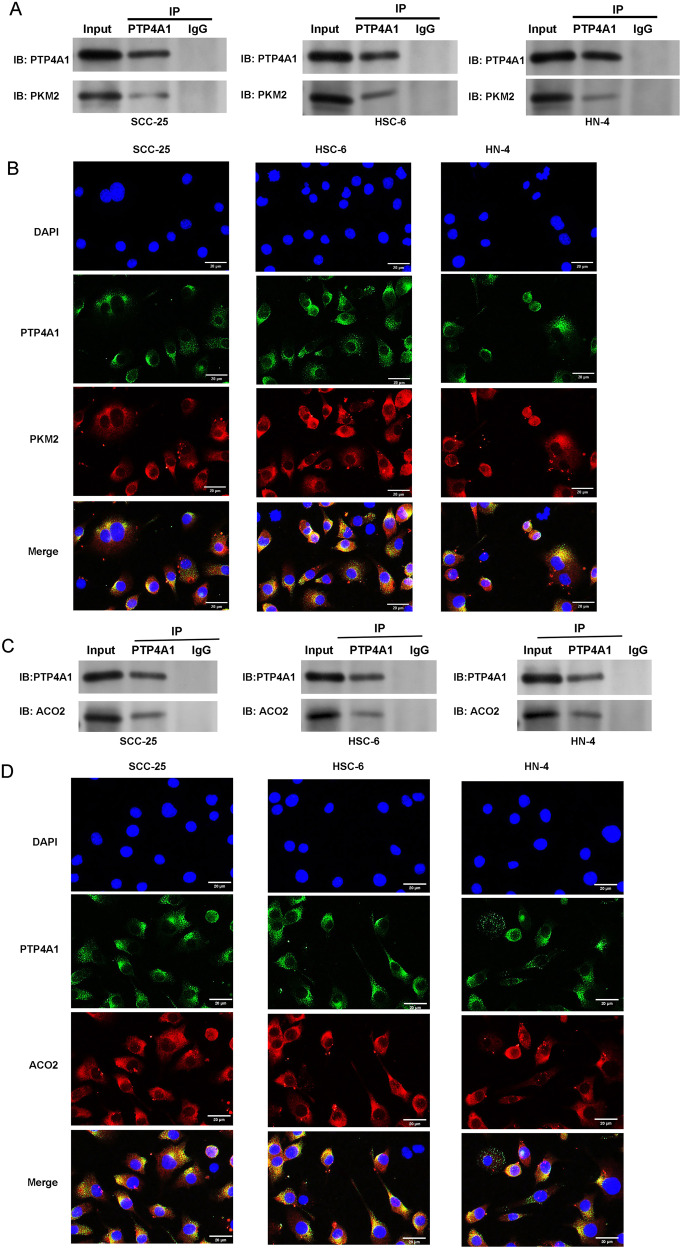
PTP4A1 associates with PKM2 and ACO2 in OSCC cells. **A** Co-IPs were performed using anti-Flag (PTP4A1) or anti-HA (PKM2) antibodies and confirmed that PTP4A1 and PKM2 interact. **B** OSCC cells stably expressing Flag-PTP4A1 were fixed for immunofluorescence analysis. PTP4A1 was detected using anti-Flag primary antibodies and Alexa Fluor 488 goat anti-mouse antibodies. PKM2 was detected using anti-PKM2 primary antibodies and Alexa Fluor 594 goat anti-rabbit antibodies. Representative cells from the same field for each experimental group are shown. **C** Co-IPs using anti-Flag (PTP4A1) or anti-HA (ACO2) antibodies confirmed that PTP4A1 and ACO2 interact. **D** OSCC cells stably expressing Flag-PTP4A1 were fixed for immunofluorescence analysis and PTP4A1 and ACO2.

Seahorse assays were used to determine energy production, glycolysis and oxidative phosphorylation in OSCC cells (SCC-25, HSC-6, HN-4) over an 80 min experimental period. sh-PTP4A1-transfected cells displayed increased OCR vs. the sh-NC group, indicative of greater levels of oxidative phosphorylation (Fig. 3A). Conversely, ECAR decreased in the sh-PTP4A1 group compared to the sh-NC group, indicating reduced the glycolytic capacity (Fig. 3B). The expression of key glycolytic (phosphofructokinase 1 (PFK1), hexokinase 2 (HK2), and PKM2) and oxidative phosphorylation enzymes (ACO2, succinate dehydrogenase (SDH) and cytochrome b (CYTB)) in OSCC cells were assessed by western blotting following sh-PTP4A1 transfection. As shown in Fig. 3C, sh-PTP4A1 transfection decreased PKM2 expression, but increased the expression of ACO2 in OSCC cells (*P* < 0.001). No changes between the sh-PTP4A1 and sh-NC groups regarding the expression of PFK1, HK2, SDH and CYTB were observed. Co-Immunoprecipitation (Co-IP) analysis in cells transfected with HA-tagged PKM2, ACO2 and Flag-tagged PTP4A1 revealed that these proteins interaction (Fig. 4A, C) and co-localization was confirmed through immunofluorescence staining (Fig. 4B, D). Collectively, these data show that PTP4A1 associates with PKM2 and ACO2 in OSCC cells.

### PTP4A1 enhances the transcription of PKM2

**Fig. 5:**
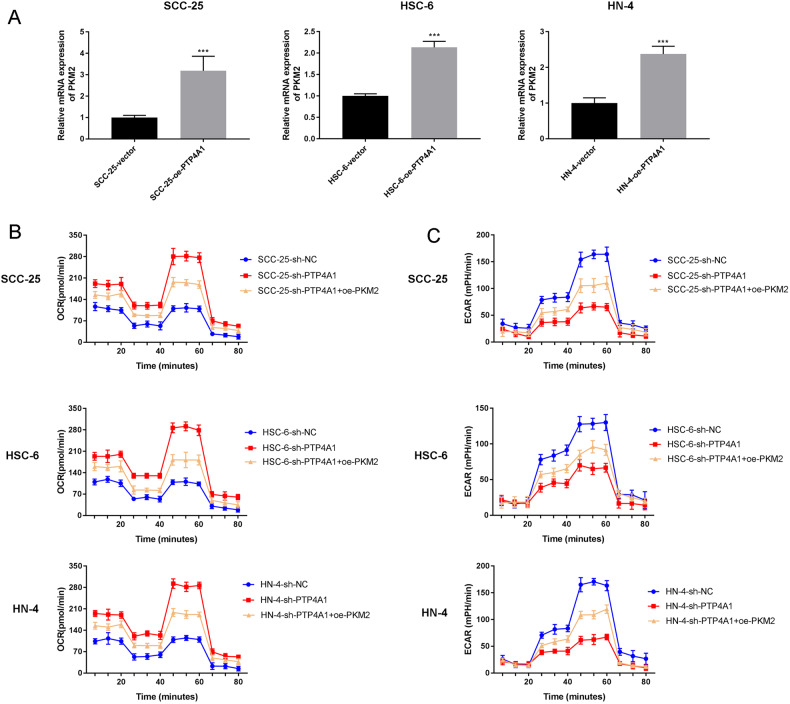
PTP4A1 participates in mitochondrial metabolic reprogramming by promoting the transcription of PKM2 in OSCC cells. **A** mRNA expression of PKM2 in OSCC cells transfected with PTP4A1 measured by RT-qPC. **B** Seahorse assays showing the oxidative consumption rate (OCR) in OSCC cells co-transfected with sh-PTP4A1 and PKM2. **C** Seahorse assays demonstrating the extracellular acidification rate (ECAR) in OSCC cells co-transfected with sh-PTP4A1 and PKM2. ***P < 0.001 vs. sh-NC.

The effects of PTP4A1 on PKM2 were further confirmed via qRT-PCR in which the relative expression of PKM2 increased in cells expressing PTP4A1 (*P* < 0.001, Fig. 5A). To investigate whether the effects of PTP4A1 on mitochondrial reprogramming were mediated by PKM2, OSCC cells were co-transfected with sh-PTP4A1 and PKM2 overexpression plasmids. Seahorse assays showed that sh-PTP4A1 transfected cells displayed significant increased OCR, which was reversed by PKM2 overexpression (Fig. 5B). Conversely, ECAR decreased in sh-PTP4A1 compared to sh-NC cells, but increased in sh-PTP4A1 + PKM2 cells (Fig. 5C). These data suggest that PTP4A1 participates in mitochondrial metabolic reprogramming through the regulation of PKM2 in OSCC cells.

### PTP4A1 promotes OSCC progression through PKM2

**Fig. 6:**
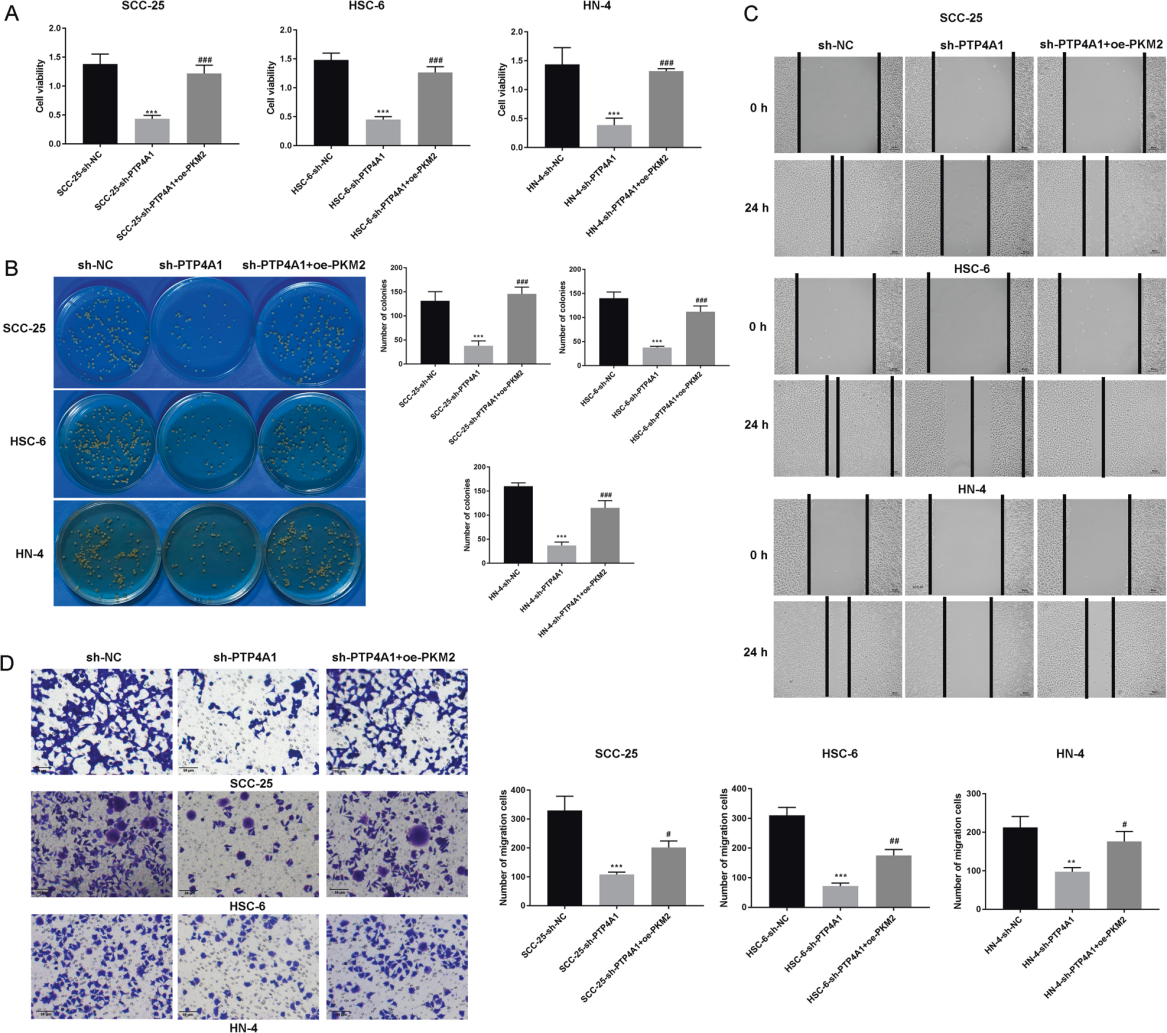
PTP4A1 participates in the growth and metastasis of OSCC cells through the regulation of PKM2. **A** MTT assays were performed to detect the viability of SCC-25, HSC-6, and HN-4 cells following co-transfection of sh-PTP4A1 and PKM2. **B** Colony formation assays. **C** Wound healing assays. **D** Transwell invasion assays. ***P < 0.001 vs. sh-NC, ^#^*P* < 0.05, ^##^*P* < 0.01, ^###^*P* < 0.001 vs. sh-PTP4A1.

OSCC cells were co-transfected with sh-PTP4A1 and PKM2 to investigate whether PKM2 could restore the metastatic phenotypes of OSCC cancer cells silenced for PTP4A1 expression. MTT- (Fig. 6A), colony formation- (Fig. 6B), wound healing- (Fig. 6C) and Transwell assays (Fig. 6D) showed that PKM2 overexpression prevented the loss of cell proliferation, migration, and invasiveness of OSCC cells that occurred as a result of PTP4A1 silencing, respectively (*P* < 0.05; *P* < 0.01; *P* < 0.001). These data confirm that PTP4A1 promotes the proliferation and metastasis of OSCC cells through regulating the expression of PKM2.

### PTP4A1 regulates the stability of ACO2

**Fig. 7:**
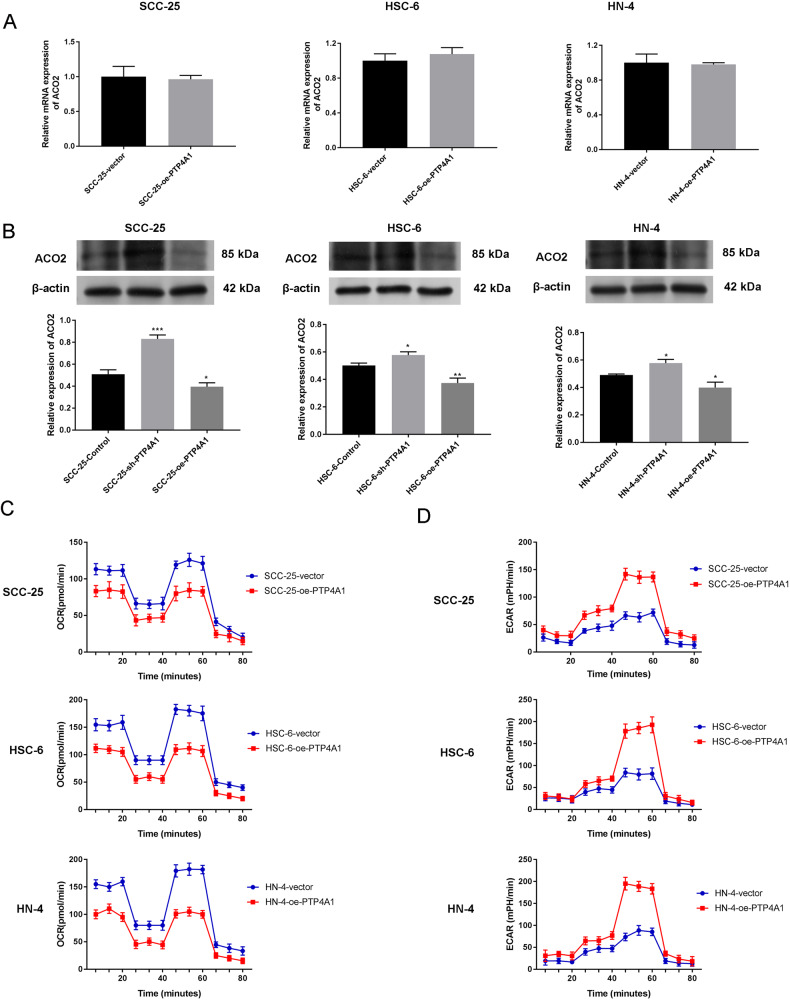
PTP4A1 participates in mitochondrial metabolic reprogramming by promoting the degradation of ACO2 in OSCC cells. **A** mRNA expression of ACO2 in OSCC cells transfected with PTP4A1 measured via RT-qPCR. **B** ACO2 expression in OSCC cells transfected with sh-PTP4A1 or PTP4A1 measured via western blotting. **C** Seahorse assays showing the oxidative consumption rate (OCR) in OSCC cells transfected with PTP4A1. **D** Seahorse assays demonstrating extracellular acidification rates (ECAR) in OSCC cells transfected with PTP4A1. *P < 0.05, **P < 0.01, ***P < 0.001 vs. Control.

Following PTP4A1 overexpression, no differences in ACO2 mRNA expression were observed (*P* > 0.05, Fig. 7A). As shown in Fig. 7B, compared to the control group, sh-PTP4A1 cells showed higher levels of ACO2 expression that decreased following exogenous PTP4A1 overexpression (*P* < 0.05, *P* < 0.01, *P* < 0.001). Seahorse assays showed that PTP4A1 overexpressed cells displayed decreased OCR and increased ECAR compared to vector only controls (Fig. 7C, D). Collectively, these data suggest that PTP4A1 participates in mitochondrial metabolic reprogramming by promoting the degradation of ACO2 in OSCC cells as opposed to effects at the transcriptional level.

### PTP4A1 promotes tumor growth in OSCC in vivo

**Fig. 8:**
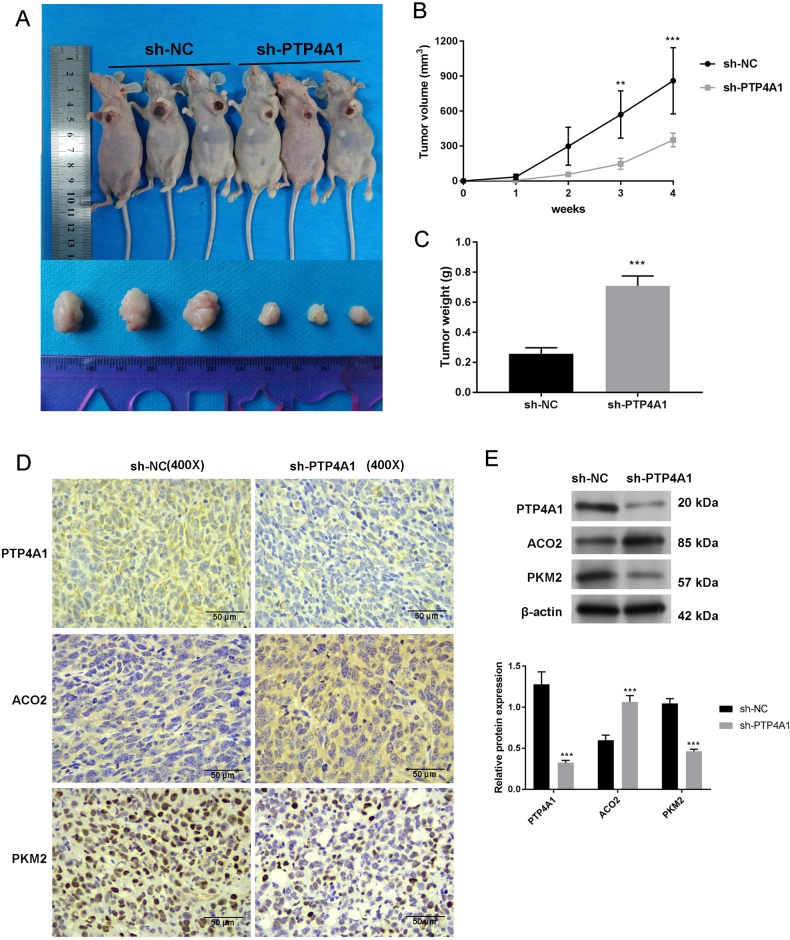
PTP4A1 knockdown suppresses the growth of OSCC xenograft tumors in vivo. **A** Effects of sh-PTP4A1 on tumor growth in xenograft models. **B** Tumor volumes. **C** Tumor weights at week 4. **D** Effects of sh-PTP4A1 on TPT4A1, ACO2, and PKM2 expression by IHC (scar bar = 50 μm, 400×). **E** Western blotting of TPT4A1, ACO2, and PKM2 in tumor tissue. **P < 0.01, ***P < 0.001 vs. sh-NC.

To confirm that the inhibition of PTP4A1 prevents OSCC tumor growth and metastasis, we implanted OSCC-25 cells expressing sh-PTP4A1 into mice and compared tumor growth. PTP4A1 silencing was found to strongly suppress tumor progression (Fig. 8A), volume (Fig. 8B) and weight (Fig. 8C) compared to sh-NC cells (*P* < 0.01, and *P* < 0.001). PTP4A1 knockdowns were confirmed by IHC, in which sh-PTP4A1 cells also showed reduced PKM2 expression and increased ACO2 expression (Fig. 8D). Western blotting analysis of the xenograft tumor tissue revealed identical results (*P* < 0.001; Fig. 8E). These data indicate that PTP4A1 knockdown attenuates xenograft tumor growth of OSCC in vivo.

## Discussion

OSCC accounts for 95% of all head and neck cancers and represents a major burden to human health. The 5-year survival rates of OSCC are low and recurrence following surgery, chemotherapy and radiotherapy remain high [13, 14]. Improved understandings of the molecular mechanisms that contribute to OSCC are required for the development of novel diagnostics and therapeutic approaches. In this study, we show that PTP4A1 is an oncogenic gene in OSCC, as its in vitro and in vivo silencing with sh-PTP4A1 suppressed the metastasis of OSCC. We further found that after sh-PTP4A1 transfection, the metabolic reprogramming of OSCC cells was altered, with decreased glycolysis and increased oxidative phosphorylation observed. These effects were attributed to PTP4A1 binding to- and activating the key glycolytic enzyme PKM2, whilst destabilizing the oxidative phosphorylation associated enzyme ACO2, most likely through enhancing its degradation. We therefore reveal new roles for PTP4A1 in the metastasis of OSCC and mitochondrial metabolic reprogramming.

PTP4A1 has been implicated in a plethora of pro-oncogenic and pathological processes [5]. Numerous lines of evidence are presented in this study to highlight its role as an oncogene in OSCC. PTP4A1 was found to be overexpressed in OSCC tissue and cell lines consistent with previous studies in ovarian carcinoma [15], intrahepatic cholangiocarcinoma [5], and hepatocellular carcinoma [16]. PTP4A1 knockdown suppressed OSCC growth, colony-forming capacity, invasion and migration. In contrast, PTP4A1 overexpression enhanced these processes consistent with previous studies in kidney [17] and intrahepatic cholangiocarcinoma [5]. PTP4A1 has reported pro-metastatic activity in OSCC cells through its ability to regulate filamentous action dynamics [18], enhanced matrix metalloproteinase expression [19], and PI3K/AKT signaling in cancer cells, thereby promoting epithelial-mesenchymal transition (EMT) processes [5]. Whether the mechanism(s) by which PTP4A1 promotes cancer cell growth differ according to cancer type remain undefined.

The tumor microenvironment (TME) can promote cancer invasion and drug resistance [11]. It is now accepted that cancer is a metabolic disease with dysregulated cellular energetics and glycolysis. Cancer cells divert energy metabolism from oxidative- towards glycolytic metabolism even under high oxygen conditions [12]. Understanding this control can open up new avenues for cancer treatment. We found that glycolysis decreases-, whilst oxidative phosphorylation increases following PTP4A1 knockdown in OSCC cells, suggestive of its capacity to induce mitochondrial metabolic reprogramming.

PKM2 is overexpressed in many tumors [20, 21]. PKM2 is the rate-limiting enzyme in glycolysis and catalyzes phosphoenolpyruvate (PEP) synthesis to promote pyruvate production [22]. Of interest, PKM2 has been shown to enhance the invasiveness of OSCC cells [23]. Here, we found that sh-PTP4A1 inhibited PKM2 expression in OSCC cells and that PTP4A1 participates in mitochondrial metabolic reprogramming through the enhancement of PKM2 expression. ACO2 is a mitochondrial enzyme that catalyzes the conversion of citrate during the tricarboxylic acid cycle [24]. Low levels of ACO2 expression are associated with poor prognosis in gastric cancer [25]. We found that sh-PTP4A1 cells show high ACO2 levels and that PTP4A1 suppresses ACO2 expression most likely through the enhancement of its degradation. Together, these findings reveal new roles for PTP4A1 during mitochondrial metabolic reprogramming in cancer.

In conclusion, our findings suggest that PTP4A1 is highly expressed in OSCC and plays an important role in OSCC development. The functional role of PTP4A1 was through mitochondrial metabolic reprogramming mediated by two pivotal proteins PKM2 and ACO2.

## Materials and Methods

### OSCC tissue

OSCC and normal (adjacent) tissues were obtained from six patients undergoing surgery from 2019 to 2021. All study protocols were approved by our internal ethics committee of our hospital and the six subjects provided written informed consent. The study was performed in accordance with the Declaration of Helsinki.

### Cell culture and transfection

Human oral keratinocyte (HOK) and OSCC cells (SCC-25, HSC-6, HN-4) were purchased from ATCC (Manassas, VA). Cells were grown in DMEM plus 10% FBS in a 5% CO_2_, 37 °C incubator. Cells were transfected with specific short hairpin RNA (shRNA) targeting PTP4A1 (sh-PTP4A1), or shRNA negative controls (sh-NC) using Lipofectamine 3000 (Invitrogen Life Technologies, USA). PTP4A1 and PKM2 plasmids were cloned into pcDNA3.1 for overexpression studies (Invitrogen, Carlsbad, CA, USA).

### Immunohistochemistry

PTP4A1 immunohistochemistry (IHC) was performed using commercial staining kits (ab269452, Abcam). Tissues were fixed in 4% PFA, paraffin embedded and sectioned (~5 μm). Sections were probed with anti-PTP4A1, anti-PKM2, and anti-ACO2 primary antibodies in 10% rabbit serum. Sections were then washed and labeled for 2 h in goat anti-rabbit IgG HRP-conjugated secondary antibodies (1:1000, ab6721, Abcam, UK). Nuclei were counterstained with hematoxylin. Cells were imaged on a DP80, Olympus microscope (Tokyo, Japan) and staining quantified at × 400 fields of view.

### RNA extraction and RT-PCR

Cells were lysed using TRIzol (Invitrogen) containing DNase-I. cDNA synthesis was performed using M-MuLV reverse transcriptase (Fermentas). Primer sequences were as follows: PKM2 (F: 5′-GCCACCATGTCGAAGCCCCATA-3’), R: 5’-TCACGGCACAGGAACAACACGC-3’), ACO2 (F: 5’-CAAATGGACGCTGTGGAAAA-3’, R: 5’-ATGGCGGAGGAAGAAGGTACT-3’), glyceraldehyde phosphate dehydrogenase (GAPDH: F: 5’-ATTCAACGGCACAGTCAAGG-3’, R: 5’-GCAGAAGGGGCGGAGATGA-3’). qRT-PCRs were performed using QuantiTect SYBR Green kits (Qiagen) in triplicate. Values were normalized to GAPDH as a reference control.

### Western blotting

Cells and tissues were lysed in RIPA buffer and subject to SDS-PAGE electrophoresis. Resolved proteins transferred to nitrocellulose membranes, blocked in 10% milk and probed with anti-PTP4A1, anti-PFK1, anti-HK2, anti-PKM2, anti-ACO2, anti-SDH, and anti-CYTB antibodies at 4 °C overnight. Membranes were washed and labeled with anti-mouse HRP-conjugated antibodies for 1 h. Proteins were visualized using the ECL system (SC-2048, Santa Cruz Biotechnology, California, USA).

### MTT assays

Cells were seeded into 96 well plates for 48 h and treated with MTT reagent. Absorbances (490 nm) were read on a spectrophotometer (Thermo Fisher Scientific, Inc.).

### Colony formation assays

Cells were grown for 2 weeks under standard culture conditions and fixed in 4% PFA. Cells were stained with crystal violet (0.1%) for 30 min and colonies counted on an inverted microscope (3 images per-sample).

### Transwell assays

Cells were plated into the upper chambers of transwell inserts in serum free media. Media plus 10% FBS was added to the lower chambers. After 48 h, cells were fixed in 4% PFA, stained with crystal violet (0.4%) and imaged. Matrigel was added into the upper chambers prior to cell seeding for invasion assays.

### Wound healing scratch assays

Cells were seeded into monolayers in serum free media for 24 h and subject to a single scratch wound using a pipette tip. Migrating cells were imaged under an inverted microscope at 0 and 24 h to quantify wound widths.

### Metabolic parameters

Cells were incubated in commercial seahorse XF assay medium plus pyruvate (1 mM), glucose (10 mM) and glutamine (2 mM) t 37 °C for 1 h in a CO_2_ free incubator. Oxygen consumption rate (OCR) and extracellular acidification rates (ECAR) and were then measured before and after oligomycin, glucose, and 2-deoxy-D-glucose (2DG) addition. FCCP (mitochondrial uncoupling agent), oligomycin (ATP synthase inhibitor), 2-DG (to inhibit glycolysis), rotenone and antimycin A were added and metabolic energy consumption assayed on a Seahorse XF96 Analyzer (Agilent, Santa Clara, CA, USA).

### Co-immunoprecipitation analysis

Cells were resuspended in BC300 buffer and sonicated on ice for 10 min. Lysates were incubated with primary antibodies (anti-PKM2, ab85555, Abcam) overnight at 4 ˚C. Anti-Rabbit IgG antibodies were included as a negative control. Agarose beads (A/G, Cell Signaling Technology) were added to the lysates for 3 h, pelleted and washed with BC300 buffer. Samples were subsequently boiled and resolved on 15% SDS-PAGE gels for western blot analysis.

### Immunofluorescence

Transfected cells were fixed, permeabilized using Triton-X-100 and blocked in 10% goat serum in PBS for 1 h. Cells were probed with rabbit anti-PTP4A1, anti-ACO2 and anti-PKM2 antibodies at 4 ˚C overnight, washed and stained with Alexa Fluor 488 donkey anti-rabbit IgG (Invitrogen, 1:1,000) for 2 h in the dark. Nuclei were counterstained with DAPI.

### Animals study and grouping

Animal experiments conformed to NIH Guidelines and were approved by our internal Ethics Committee of our hospital. BALB/c nude mice (6 weeks, 22–25 g, Charles River, China) were fed a defined diet and provided water *ad libitum*. OSCC cells expressing sh-PTP4A1 were resuspended in PBS (2 × 10^6^, 150 µL), and subcutaneously injected into mice flanks. Virus supernatants (5 × 10^8^ pfu) were intratumorally administered twice per-week. The size and volumes of the tumors were measured with a caliper and analyzed using the following formula: length × width^2^ × 0.5^2^. After 4 weeks, mice were sacrificed with sodium pentobarbital (40 mg/kg, i.p.). Excised tumors were weighed and subjected to western blotting and IHC analyses.

### Statistical analysis

Data analysis was performed using SPSS 20.2. Values represent the mean ± SD from 3 independent experiments. A student’s (two-tailed) t-test or one-way analysis of variance (ANOVA) were performed for single or multiple group comparisons, respectively. *P* < 0.05 was deemed significant.

### Supplementary information

Supplementary Information

## Data Availability

All the data used to support the findings of this study are included within the article.
