# Supplementary figures and images for: PTP4A1 promotes oral squamous cell carcinoma (OSCC) metastasis through altered mitochondrial metabolic reprogramming
Source: Cell Death Discov. 2023 Sep 29;9:360. doi: 10.1038/s41420-023-01657-x (PMC10541904; doi:10.1038/s41420-023-01657-x)

Figure 1B

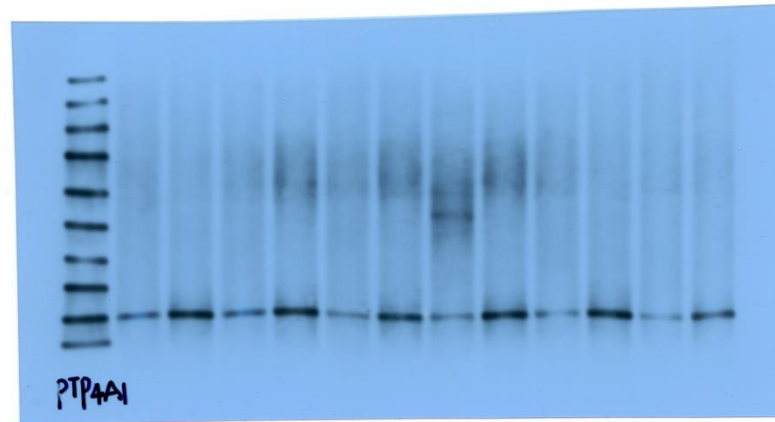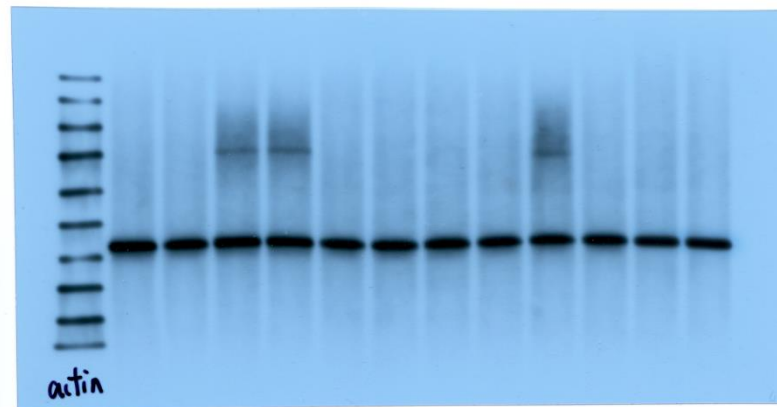

Figure 1C

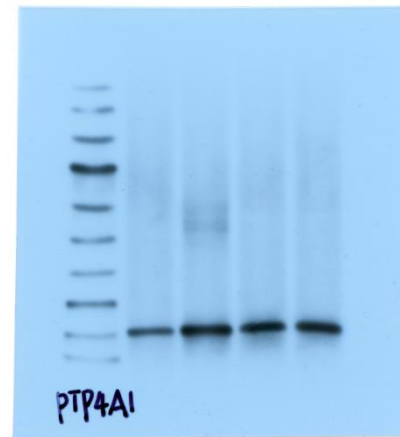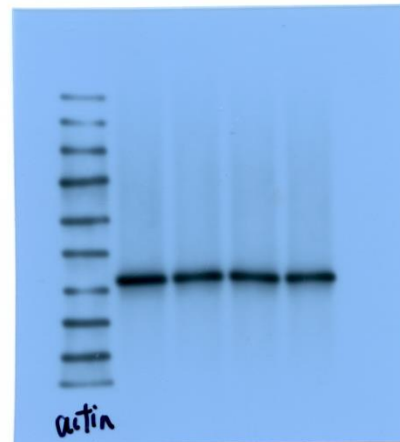

Figure 2A

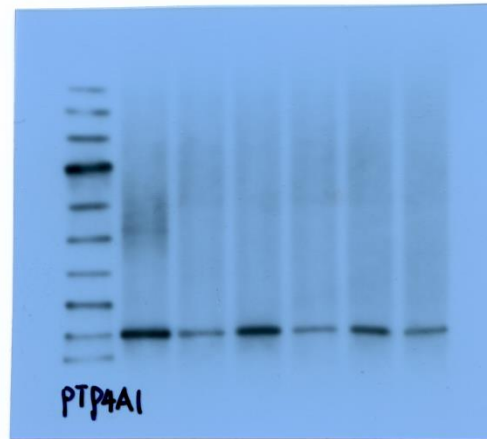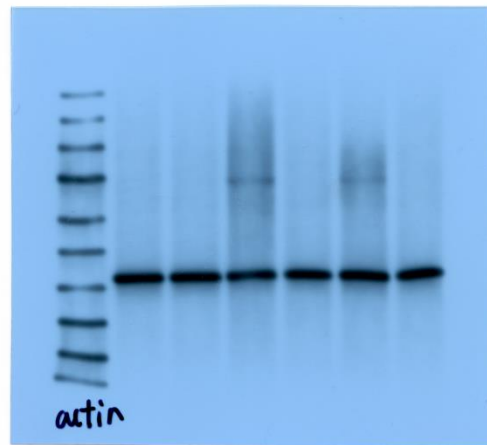

Figure 3C

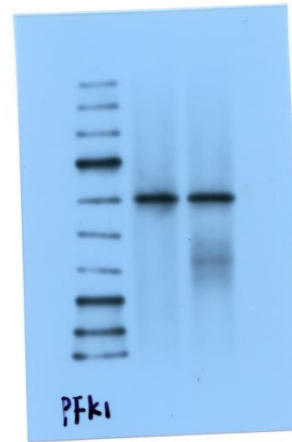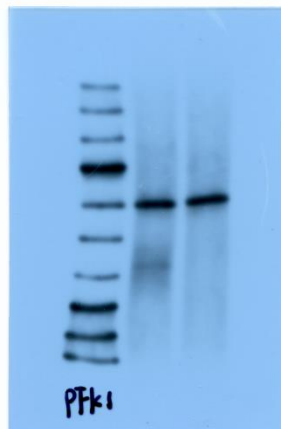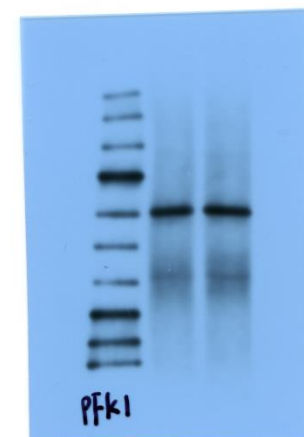

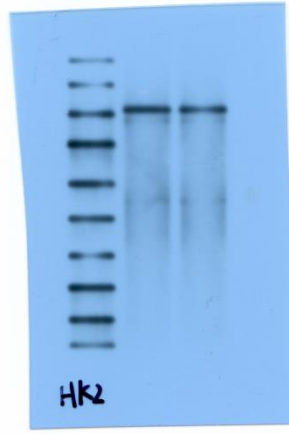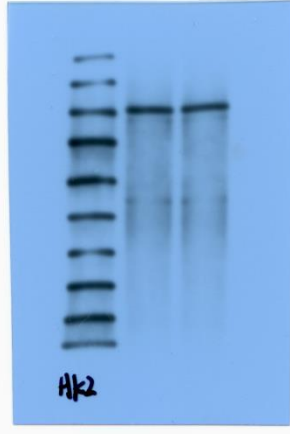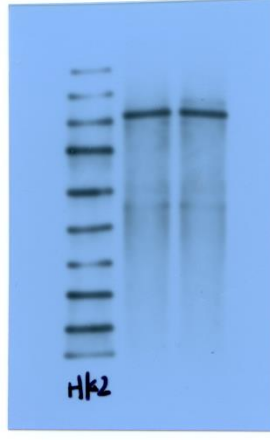

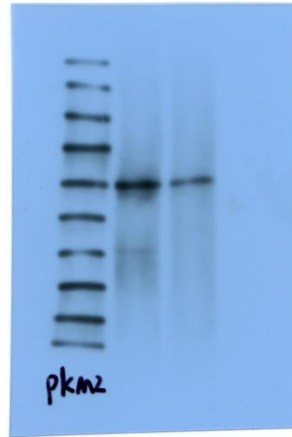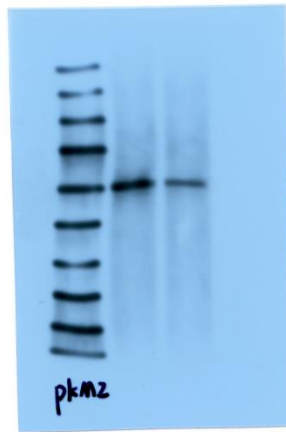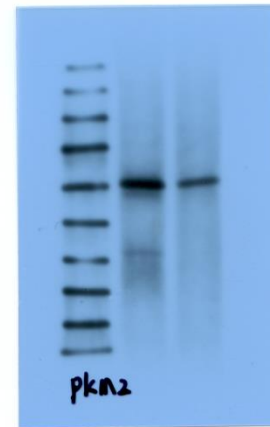

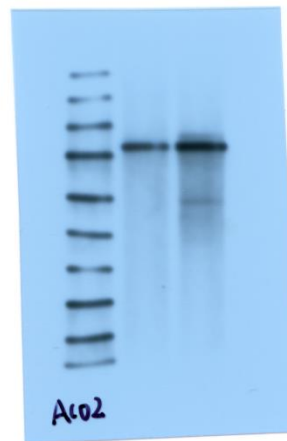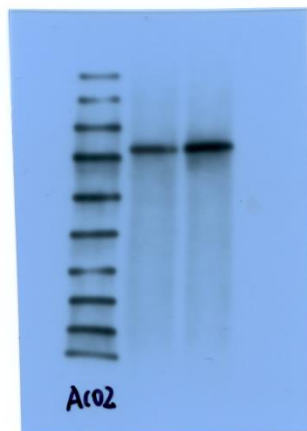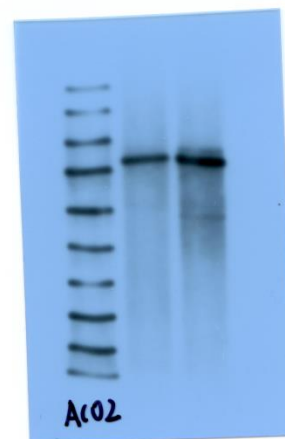

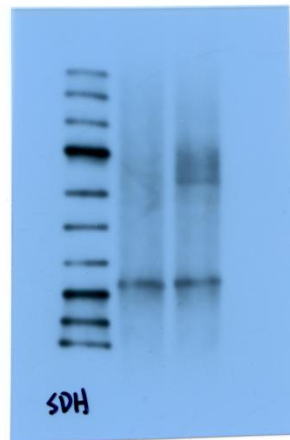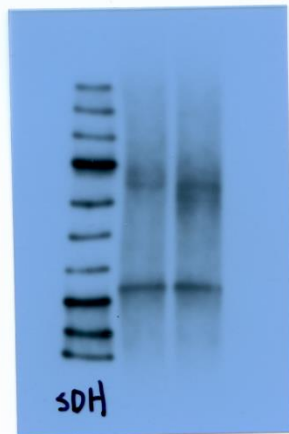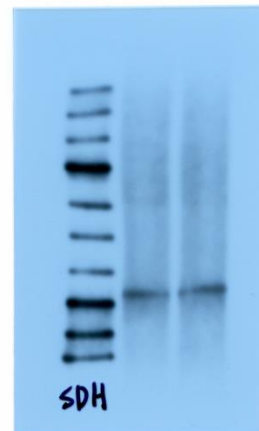

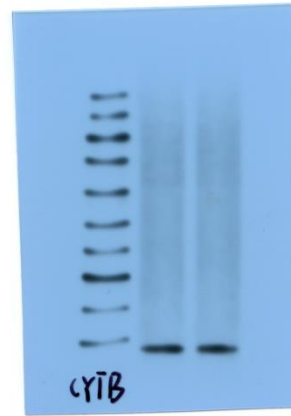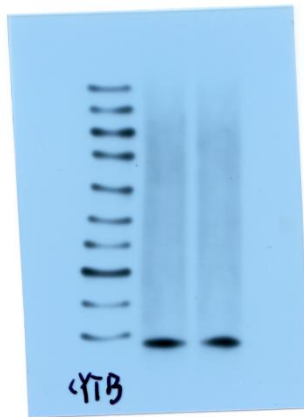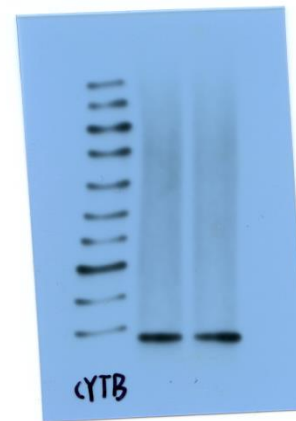

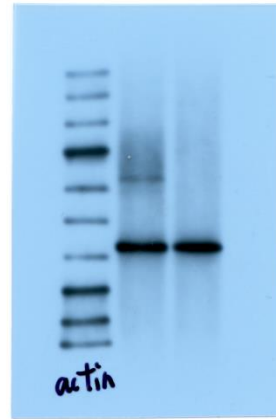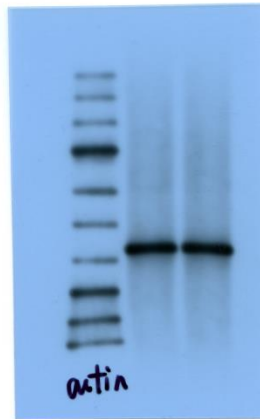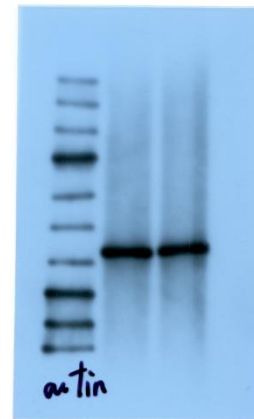

Figure 4A

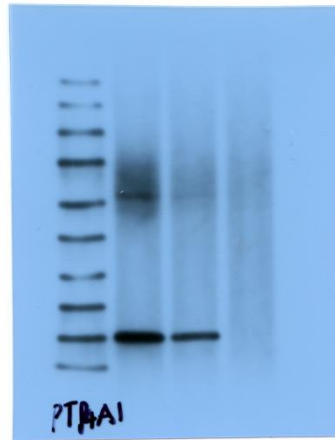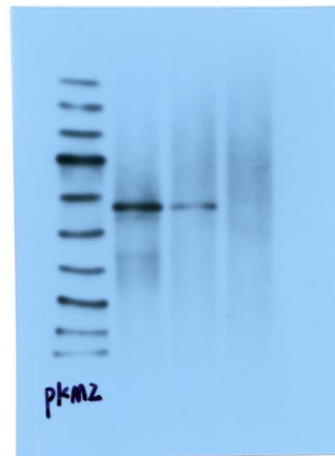

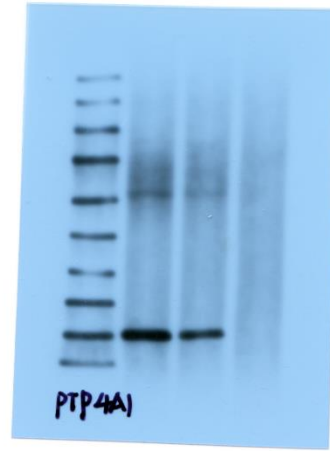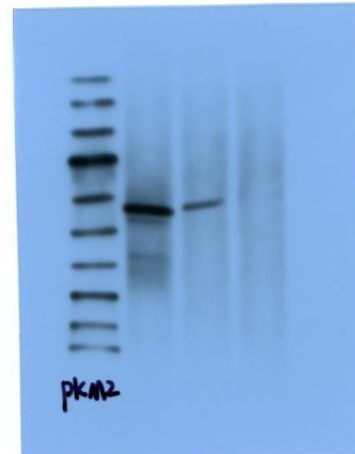

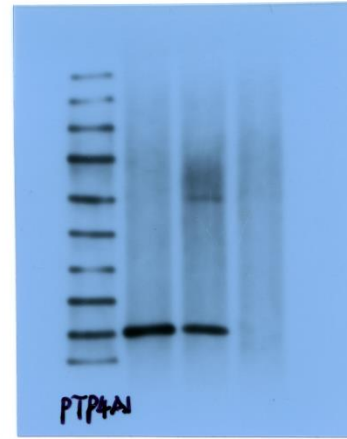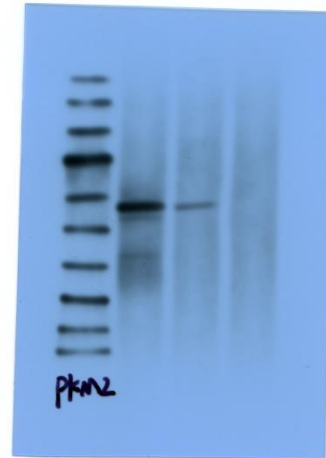

Figure 4C

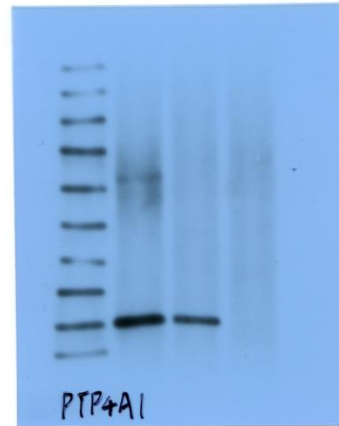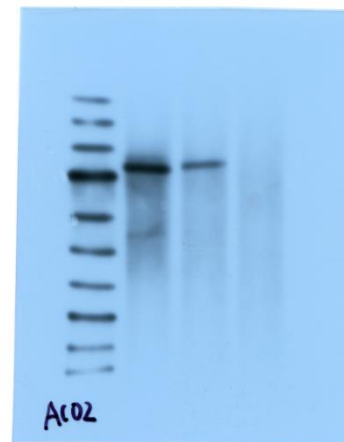

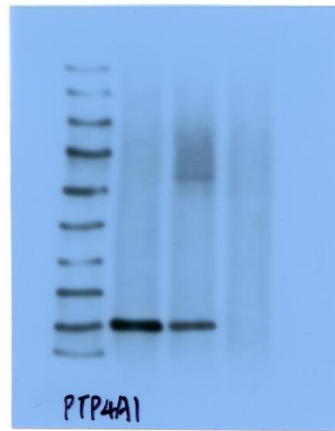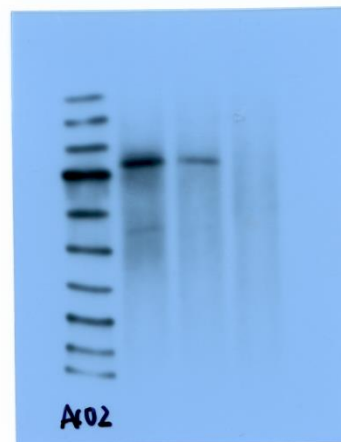

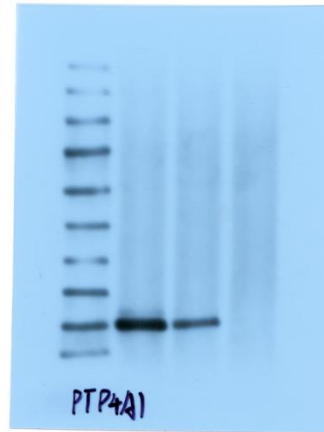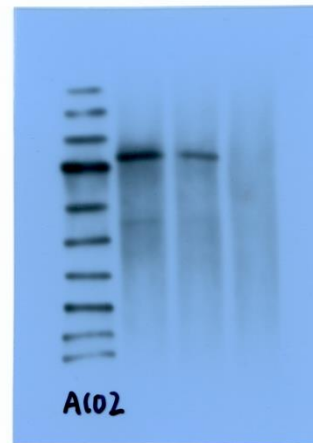

Figure 7B

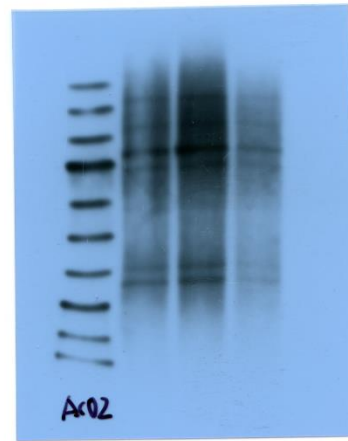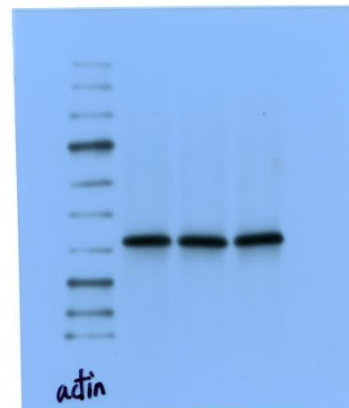

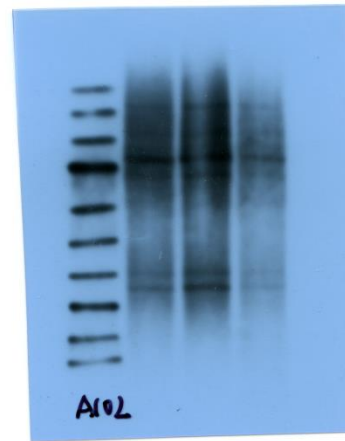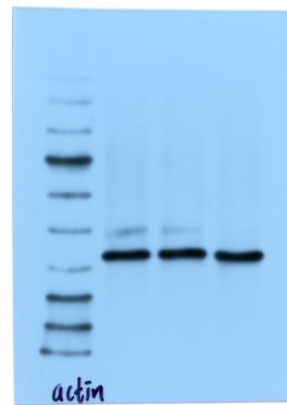

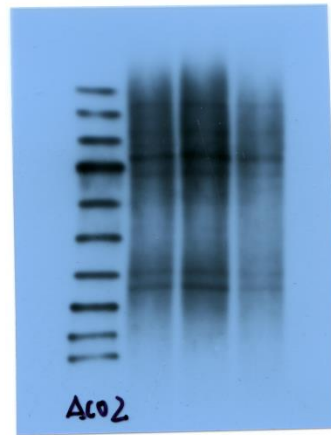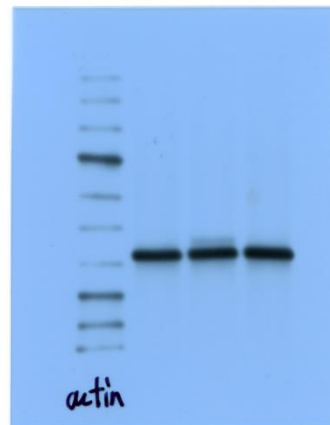

Figure 8E

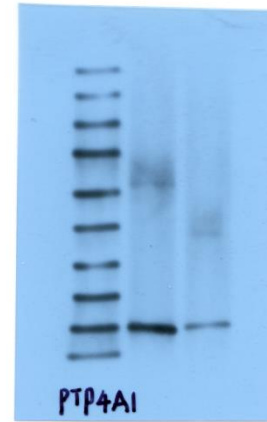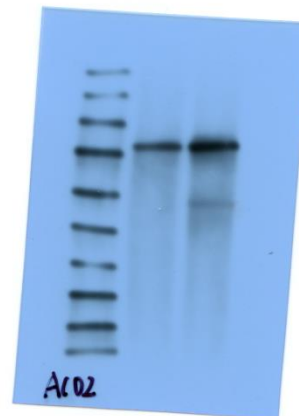

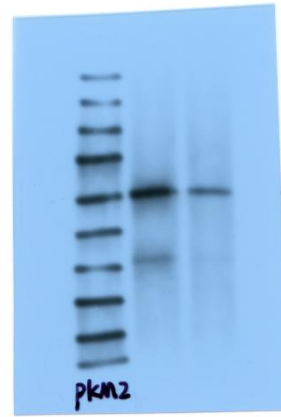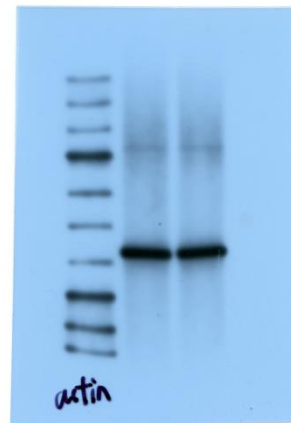

Supplement: Supplementary file 1 — Supplementary Information [file 41420_2023_1657_MOESM1_ESM.pdf]
